# Effects of dietary gluten on body weight and gut microbiota in BALB-C mice using 16 S rRNA-Based analysis

**DOI:** 10.1038/s41598-025-92213-3

**Published:** 2025-03-07

**Authors:** Merve Sayın Dülger, Nihal Zekiye Erdem, Emek Dümen

**Affiliations:** 1 Institute of Health Sciences, Department of Nutrition and Dietetic, Istanbul Medipol University, Göztepe Mahallesi, Atatürk Caddesi. No: 40/16, 34815 Beykoz, İstanbul, Türkiye; 2 College of Health Sciences, Department of Nutrition and Dietetic, Istanbul Medipol University, Cibali Mahallesi, Unkapanı, Atatürk Bulvarı, No: 27, 34083 Fatih, İstanbul, Türkiye; 3 School of Veterinary Medicine, Department of Food Hygiene& Technology, Istanbul University Cerrahpaşa, Alkent 2000. Mahallesi, Yiğittürk Caddesi, Avcılar, İstanbul, Türkiye

**Keywords:** Nutrition, Gluten, Microbiota, Body weight, High-fat diet, Microbiota, Nutrition

## Abstract

Despite the widespread adoption of gluten-free diets for weight management, the relationship between gluten intake and obesity remains unclear because of the limited number of controlled studies available in the literature. Furthermore, there is ongoing debate regarding the impact of gluten-containing diets on the gut microbiota. This study aimed to investigate the effects of gluten consumption on the body weight and intestinal microbiota of mice fed a high-fat diet. Twenty-four Bagg albino laboratory-bred mice (BALB/c) were randomly divided into four groups for oral gavage feeding: standard diet control (SDC), standard diet + 5 mg/day gluten (SD + gluten), high-fat diet control (HFDC), and high-fat diet + 5 mg/day gluten (HFD + gluten). Each subject’s body weight was measured and recorded weekly. For microbiota analysis, fecal samples were collected weekly from the cages after overnight cage changes. The microbiota was analyzed using via the 16 S ribosomal ribonucleic acid (rRNA) method. Compared with the control diet, both gluten consumption and a high fat diet significantly increased weight gain (*p* < 0.05). No significant difference was observed in the total mesophilic aerobic bacterial count among the groups (*p* > 0.05). However, the addition of gluten to the diet positively affected *Lactobacillus bulgaricus* (*p* < 0.05). Conversely, gluten-containing diets negatively impacted the total coliform bacteria and *Escherichia coli* counts in the gut (*p* < 0.05). These findings suggest that gluten, when combined with either a normal diet or a high-fat diet, contributes to weight gain while exerting positive effects on the intestinal microbiota.

## Introduction

Changes in the human microbiota that occur with industrialization are thought to be the underlying factor in the dramatic increase in diseases such as obesity and metabolic diseases. Microbiota describes the living microorganisms found in a defined environment, such as skin and gastrointestinal system, while the microbiome includes their genomes, metabolites and environmental factors^1,2^. Among these, the gut microbiota plays an important role in processes related to weight regulation^3,4^. Studies have shown that severe obesity is linked to reduced microbial diversity and abundance in the gut, with obese individuals having different microbiota compositions compared to lean individuals^5,6^. Obesity-associated microbiota may contribute to weight gain by increasing the production of short-chain fatty acids (SCFAs), such as butyrate, which provide extra energy as a result contributing to obesity^7^. The macro- and micronutrient contents of the diet are recognized as critical factors in shaping the gut microbiota^8^. However, the specific effects of protein intake from different dietary sources on obesity development and the composition of the gut microbiota are not fully understood^9^. Wheat gluten is considered a beneficial dietary protein, as it provides amino acids, such as glutamine and glutamic acid, which play a role in modulating gut immunity^10^. Previous studies have reported an increase in beneficial bacteria such as *Lactobacillus* while others have noted negative effects, including an increase in potentially pathogenic bacteria such as *Clostridium XI* alongside a decline in beneficial bacteria like *Lactobacillus*^5,10–12^. Research on celiac disease patients has shown that a gluten-free diet can lead to reduction in *Lactobacillus* while increase pathogens like *Escherichia coli*^12,13^. Gluten-free diets have been employed as potential strategies for antiobesity, anti-inflammatory, and antidiabetic effects^10^. Nevertheless, it remains unclear whether the effect of dietary gluten on body weight results from its direct impact on adipose tissue or from systemic inflammation triggered by its presence in the gastrointestinal system^14^. Animal studies have shown that gluten supplementation in high-fat diets may lead to obesity by reducing thermogenesis and browning of subcutaneous fat tissue and increasing adipocyte size^11,15^. In addition, studies in humans have shown that low-gluten diets are associated with increased thermogenesis due to increased levels of peptide YY and beta-aminoisobutyric acid (BAIBA) leading to weight loss^12^. Evidence also suggests that gluten’s influence on gut microbiota or its direct effects on intestinal barrier integrity may contribute to inflammation and subsequent weight gain. Gliadin, a gluten component, has been reported to trigger the release of zonulin, a protein that disrupts tight junctions like occludin and E-cadherin, leading to increased intestinal permeability^10^.

This study aimed to investigate the effects of gluten consumption and high-fat diets on body weight and gut microbiota composition using the BALB-C mouse model by 16 S ribosomal RNA-based analysis. While many previous studies focused on disease-specific conditions (such as celiac disease, prediabetic mice) and used gliadin alone, which is considered allergenic in gluten^5,16^, this study aimed to better isolate the effects of dietary interventions without any disease physiology.

## Materials and methods

### Research site and sampling selection

This study was conducted at the Istanbul Medipol University Medical Research Center (MEDITAM) from November 2019 to October 2020. All authors comply with the ARRIVE guidelines, and all methods were performed in accordance with the relevant guidelines and regulations. Male BALB/c mice were obtained from the MEDİTAM. The research involved twenty-four male BALB/c mice, aged four weeks, with an average weight of 20–30 g, distributed homogeneously. Ethical approval for the study was obtained from the Istanbul Medipol University Animal Experiments Local Ethics Committee (Approval date: 30/09/2019, Approval number: 62).

### General plan of the study

After one week of acclimatization, mice were randomized into four groups (*n* = 6) based on the initial body weights and placed into four cages, with six mice in each cage (*n* = 6): standard diet control (SDC), standard diet + 5 mg/day gluten (SD + gluten), high-fat diet control (HFDC), and high-fat diet + 5 mg/day gluten (HFD + gluten). During the study period, the mice were fed *ad libitum *at a constant room temperature of 21 ± 2 °C, and their natural light-dark cycles were maintained^17^. The body weights of the mice were measured and recorded prior to the initiation of the gluten and high-fat diet interventions. Sterile gluten was dissolved in acetic acid at a concentration of 5 mg/day and administered by oral gavage three times a week. Administering gavage three times a week was chosen to enhance the feasibility of dose application and minimize the stress levels induced by the gavage method in mice. The dose for our study was determined based on the literature, the estimated daily gluten intake from gluten-enriched diets used in previous studies, and human gluten consumption using the simple weight scaling method ^12,18–21^. To ensure consistency, food access was restricted one hour before gavage. In the nongluten groups, acetic acid was gavaged three times a week to simulate the same stress conditions. The body weight of each subject was monitored and recorded on a weekly basis. The cages were changed overnight, and fecal samples were collected from the cages once a week. These samples were stored in a deep freezer at −80°C until DNA extraction^9,12^.

### Anthropometric measurements

Each mouse was tagged with an identification number for body weight measurement. Body weights were measured in grams using via the Weightlab WH-2002 instrument.

### Dietary intervention

After the acclimatization period, the mice were assigned to different diet groups. The standard diet consisted of Altromin 1324 (Altromin GmbH, Lage, Germany) mouse feed. The high-fat diet was prepared by adding 19.5 g of butter to 100 g of standard laboratory feed. The butter was melted and evenly mixed into the feed, which was then roasted. The macronutrient composition of a high-fat diet, on the basis of energy content, is 65% fat, 24% protein, and 11% carbohydrates^22,23^.

### Isolation and identification procedure

After the mice were fed according to the specified procedure, changes in the diversity of *Lactobacillus*, coliform group, and *Escherichia coli* bacteria in the intestines were assessed weekly.

#### Lactobacillus spp

Isolation was performed via appropriate dilutions on Man, Rogosa, and Sharpe (MRS) agar (Merck, Germany) for lactobacilli and Nutrient Agar (NA) (Merck, Germany) for enterococci, via the spread plate method. Following 48 h of incubation at 30 ˚C for MRS agar (pH 5.7 ± 0.2) and at 37 ˚C for NA (pH 7.0 ± 0.2), colonies exhibiting different morphological characteristics were selected. For enterococci, small, white or pale-colored, smooth-edged colonies were prioritized, whereas for lactobacilli, cream-colored, matte, smooth-edged colonies were preferred. The microscopic appearance, Gram reaction, and catalase activities of the isolates were examined for purity control. Cocci-shaped, gram-positive, catalase-negative isolates of various sizes were reserved for enterococci, whereas rod-shaped, gram-positive, catalase-negative isolates were reserved for lactobacilli-specific identification tests^24^.

#### Total coliform group bacteria

The samples were collected and transported to the laboratory in sterile containers under aseptic conditions. The samples were subjected to dilution and homogenization procedures, followed by the standard spread plate method. This method was applied via use of Violet Red Bile (VRB) agar (Merck, Germany), which was previously prepared and poured into Petri dishes. After cultivation, a second layer of VRB agar was added to the Petri dishes, which were subsequently incubated at 37 °C for 24 h. The typical colonies that formed at the end of the incubation period were subsequently counted^25^.

#### Escherichia coli

Dilution and homogenization procedures were applied to the samples. The standard spread plate method was subsequently used with TBX (Tryptone Bile X-glucuronide) agar, which was subsequently poured into preprepared Petri dishes. The Petri dishes were incubated at 44 °C for 24 h, and the typical colonies that formed at the end of the incubation period were counted^26^.

## DNA extraction

The DNA of all the isolates was extracted via a commercial DNA extraction kit following the manufacturer’s protocol. The extracts were stored at −20°C for use as target DNA in PCR procedures^17^.

### PCR

The primers designed specifically for the microbiological parameters to be analyzed in this study for use in PCR procedures are presented in Table 1^17^.

*Lactobacillus spp.* parameter, *Lactobacillus acidophilus*,* Lactobacillus delbrueckii subsp. bulgaricus*, and *Lactobacillus brevis*, which are considered to have the most significant effects on obesity in this study, were included in the PCR procedure.

**Table 1:** Primer sets planned to be used in our study and their characteristics.

Primer No	Sequence (5’ – 3’)	Target Gene / Amp (bp)	Target Microorganism
1	AAGAAACTTTGTTTAGTTTTGAGGTA	*16s rRNA*	*Lactobacillus acidophilus*
2	CAATTTTCGTGTCCCCTTCG GTTAATGATAGTGTGTCGAAAC	*23 S /* 450	*Escherichia coli*
3	AAGAACTTTGTTCAGTTTTGAGAGTA	*16s rRNA*	*Lactobacillus delbrueckii subs bulgaricus*
4	TTGAAACAATGTTCAGTTTTGAGGGGC	*16s rRNA*	*Lactobacillus brevis*

(**Amp**: **Amplicon**, **bp**: **Base Pairs)**

The PCR mixture was prepared as follows: 2 µl of DNA sample, 2.5 mM MgCl₂, 10 mM Tris-HCl (pH 8.0), 5 mM KCl (0.2 mM of each nucleotide), 0.8 µmol/ml of each primer, and 1 U of Taq DNA polymerase (final volume of 25 µl). Amplification procedures were carried out according to the protocol. The initial denaturation was set at 94 °C for 5 min, followed by 35 cycles of the following steps: 1 s at 94 °C for denaturation, 1 s at 55 °C for primer annealing, and 21 s at 72 °C for elongation. A final extension step was performed at 72 °C for 7 min^17^.

### Electrophoresis

The PCR products were electrophoresed in 2% (wt/vol) agarose containing ethidium bromide, and the presence of specific bands was detected via a UV transilluminator^17^.

### Statistical analysis

For normality analysis, the Kolmogorov-Smirnov test and skewness/kurtosis measures were examined. For group comparisons, ANOVA and Welch tests were utilized in cases where parametric assumptions were met. In non-parametric situations, the Kruskal-Wallis test was employed. To explore differences between groups, LSD was applied for parametric tests, while the Mann-Whitney U test was used for non-parametric analyses. A significance level of 0.05 was adopted for all statistical tests. Blinding was ensured during the study by coding group assignments, with researchers (except the primary investigator) and the statistician unaware of group details until the analysis was completed. Blinding was ensured during the study by coding group assignments, with researchers (except the primary investigator) and the statistician unaware of group details until the analysis was completed.

To determine the appropriate sample size for this study, a power analysis was conducted. The calculations indicated that a sample size of 21 mice would be sufficient to obtain statistically significant results.

## Results

### Changes in body weight

According to the results obtained, the gluten and high-fat diet interventions caused statistically significant weight gain compared with the control group (*p* < 0.05). Both high-fat and gluten intake induced weight gain in the subjects (Table S2).

### Total mesophilic aerobic Bacteria

There was no statistically significant difference between the groups in terms of total mesophilic aerobic bacteria count (*p* > 0.05). The different dietary protocols applied had no effect on this microbiological parameter (Table S3).

### L. Bulgaricus

The addition of gluten to the diets significantly stimulated the growth of *L. bulgaricus* (*p* < 0.05) (Table S4).

### Total coliform group Bacteria

It was found that both the high-fat diet and the gluten-containing diet groups inhibited the growth of total coliform group bacteria (*p* < 0.05). No significant difference was detected between the high-fat and high-fat + gluten groups in terms of total coliform bacteria count (*p* > 0.05) (Table S5).

### E.Coli

It was found that the high-fat diet inhibited the growth of *E. coli* in the intestine (*p* < 0.05). A negative effect of the gluten-containing diet groups on total *E. coli* growth in the intestine was also observed (*p* < 0.05). No significant difference was found between the high-fat and high-fat + gluten groups in terms of *E. coli* growth (*p* > 0.05) (Table S6).

### PCR analysis

**Fig. 1:**
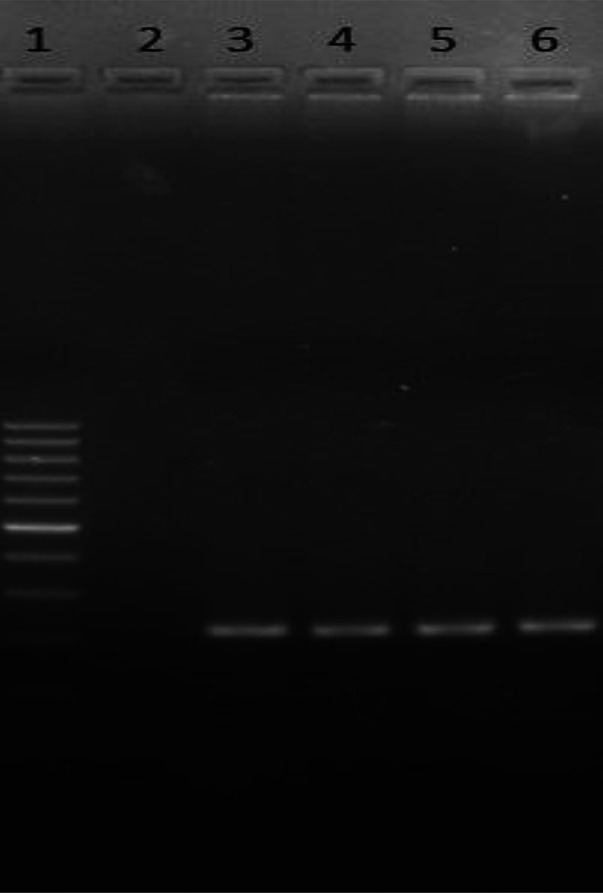
Agarose gel electrophesis image of amplified PCR products of *E. coli* strains (1-SM, 3 / 6- *E. coli* 23 S / 450, 2-negative control; SM: standard marker).

**Fig. 2:**
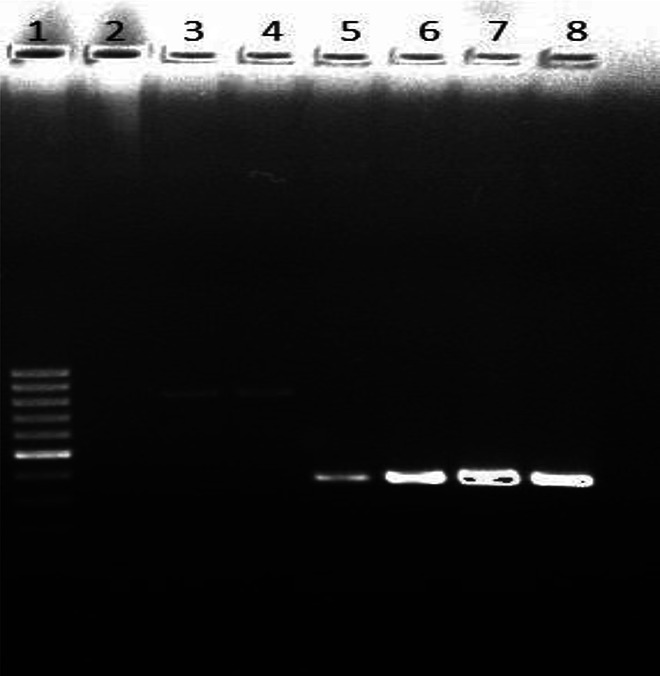
Agarose gel electrophoresis image of *Lactobacillus spp*. amplified PCR products (1-SM, 5 / 8- *L. delbrueckii subs bulgaricus* 16s rRNA, 3 / 4- *L. acidophilus* 16s rRNA, 2-negative control; SM: standard marker).

The agarose gel images obtained from the PCR analysis of the microorganisms studied are presented in Figs. 1 and 2.

## Discussion

The prevalence of gluten-free diets is increasing, with nearly one in three people in the United States attempting to eliminate gluten from their diet in the past decade. This trend is reportedly driven by the belief that gluten-free diets are beneficial for overall health and promote faster weight loss^27^. Gluten-free diets are also widely used for weight control and the treatment of intestinal diseases. However, a small number of controlled studies have reported inconsistent findings regarding the relationships among gluten, obesity, and the gut microbiota^14,28,29^. Although some studies in mice have investigated the combined effects of high-fat diets and gluten intake, conflicting findings highlight the need for further research. Additionally, many previous studies have focused on disease-specific conditions (such as celiac disease, prediabetic mice) and disease physiology is known to influence the microbiota, and some studies have used gliadin alone, which is considered an allergen in gluten^5,11,30^.

This study aimed to investigate the effects of gluten consumption and high-fat diets on body weight and gut microbiota composition using the BALB-C mouse model. The analysis was performed by 16 S ribosomal RNA-based sequencing to better isolate the effects of dietary interventions using a healthy animal model without any disease physiology. In our study, high-fat diet interventions caused statistically significant weight gain compared to the control group (*p*< 0.05). Our observations are consistent with earlier research findings. Chronic exposure to high-fat diets (HFDs) disrupts glucose homeostasis and energy metabolism, leading to increased body weight. For example, in a study in which mice were fed an HFD for 20 weeks, significant weight gain was observed^31^.

Our study revealed that gluten contributes to weight gain both independently and when combined with a high-fat diet (*p*< 0.05). These observations support the conclusions of previous studies. For instance, mice fed a Western diet containing gluten were found to exhibit the highest body weight gain, adiposity, and larger adipocyte size^11^. In the referenced study, unlike ours, the mice were fed a high-fat and high-sucrose Western diet. Sucrose is known to trigger weight gain and fat accumulation^32^. Evidence from another experiment demonstrated that mice fed a high-fat diet with added gluten showed greater body weight and reduced energy expenditure^33^. Furthermore, a study conducted in Denmark reported that a low-gluten diet led to an average weight loss compared to a high-gluten diet, despite unchanged energy intake^12^. However, caution should be exercised when comparing these findings to human studies, given the fundamental interspecies differences.

The impact of dietary gluten on body weight remains uncertain, whether due to its direct effects on adipose tissue or the systemic inflammation it triggers in the gastrointestinal system. In a mice study, reported that gluten intake leads to weight gain by decreasing browning and thermogenesis markers in subcutaneous fat^32^. Specifically, gluten consumption decreases the levels of UCP1 (uncoupling protein 1) in brown adipose tissue (BAT), a key regulator of thermogenesis, thereby lowering energy expenditure. Additionally, it suppresses the expression of BMP7, a protein that promotes the browning of white adipose tissue, further limiting energy dissipation^12,14^.

Low-gluten intake has been associated with increased concentrations of peptide YY (PYY), a hormone that suppresses appetite postprandially and beta-aminoisobutyric acid (BAIBA) which is known to increase thermogenesis by promoting the conversion of white fat tissue into brown fat tissue. These factors may help increase thermogenesis and reduce weight gain. In contrast, gluten consumption in mice has been shown to reduce oxygen consumption and energy expenditure during fasting, leading to decreased thermogenesis. Moreover, the expression of thermogenic genes was observed to decrease across all adipose tissues in gluten-fed mice, potentially explaining the observed weight gain^12,14^.

The potential impact of gluten on weight gain through its effects on the gut has been explained in several studies, suggested that gluten administration may lead to gut barrier dysfunction^5^. Furthermore, a mouse study revealed that duodenal mucosal damage and reduced expression of tight junction proteins was linked to weight gain^31^.

In our study, it was determined that gluten intervention stimulated the production of *Lactobacillus bulgaricus*, a genus thought to be beneficial for the intestinal microbiota (*p* < 0.05), and had a statistically significant inhibitory effect on the growth of *Escherichia coli* and total coliform bacteria, both of which are considered pathogens (*p* < 0.05). In addition, no statistically significant difference was detected between the groups in terms of the total mesophilic aerobic bacteria count parameter (*p* > 0.05).

In a 23-week study examining the effects of gliadin in mice fed a high-fat diet found increased opportunistic pathogens such as *Clostridium XI*,* Dorea*, and *Coriobacteriaceae*, while *Akkermansia muciniphila* (considered beneficial) was increased, but *Lactobacillus*, a beneficial genus particularly important for gut health, was significantly decreased^5^. The differences in study results may be due to the longer experimental period with gliadin instead of gluten^5^. A study investigating wheat gluten intake with a high-fat diet showed a reduction in the relative abundances of Firmicutes and Lactobacillus, but an increase in the relative abundances of Bacteroidales S24-7 and Ruminococcaceae^34^. However, the findings of this study may differ from ours, as the comparator group was fed casein. Casein is unique in that it contains a high proportion of branched-chain amino acids (BCAAs), which can affect the microbiota^35^.

Furthermore another study demonstrated that healthy bacteria, such as *Bifidobacterium* and *Lactobacillus*, were reduced, whereas unhealthy bacteria (especially *E. coli*) increased in the fecal microbiota of healthy adults following a gluten-free diet^28^. Our research revealed similar trends as those highlighted in this study. Still, we need to be careful when comparing these results to humans because of the natural differences between species.

High-fat diets are known to affect the gut microbiota. A study investigating the effects of a high-fat diet (HFD) on the gut microbiota found that *Escherichia-Shigella* was increased in the high-fat diet group, while conversely, the abundance of *Staphylococcus*,* Ralstonia*,* Vagococcus*, and *Streptococcus*was significantly reduced^36^.

Our findings are different from previous studies, as it has been determined that the high-fat diet in our study significantly reduced the growth of *Escherichia coli* and total coliform bacteria in the gut (*p*< 0.05). This difference may be due to the shorter study duration (4 weeks) or the role of high-fat diets in increasing bile acid secretion. It has been observed that higher bile acid levels lead to the production of secondary bile acids, which are known to suppress some Gram-negative pathogens. However, while *E. coli* has developed mechanisms to resist bile-induced stress, its survival may decrease under nutrient competition and environmental stress^37^.

In addition, it has been determined that there was no significant difference in the total counts of mesophilic aerobic bacteria between groups (*p* > 0.05), which may be due to the broad diversity of this bacterial group. Future studies focusing specifically on mesophilic aerobic bacteria could provide clearer information about the effects of high-fat diets.

In a study investigating the effects of prebiotics alongside the effects of gluten described in the literature, prebiotics were found to reduce fat accumulation, restore fat-burning markers, and increase beneficial bacteria which breaks down gluten peptides and reduces inflammation. Additionally, prebiotics inhibited the growth of obesity-associated bacteria, highlighting their potential to counteract the negative effects of gluten and improve gut health. Furthermore, in human diets, gluten is primarily consumed through wheat-based products, which also supply carbohydrates and prebiotics. These additional components may influence weight-related outcomes and microbiota composition, potentially affecting the observed effects^11^.

## Limitations

The primary challenges in this study included the dissolution of gluten for administration to the mice and managing the subjects’ mobility of the subjects during gavage, which hindered the complete delivery of gluten to the stomach.

## Conclusion

Our study demonstrated that gluten consumption contributes to weight gain under both normal and high-fat diet conditions. Unlike research that focuses on the effects of gluten in the context of disease states such as celiac disease, prediabetes, or colitis, we conducted our study using healthy mice. This provides a fresh perspective on how gluten affects weight and gut health in non-disease scenarios.

Interestingly, our results revealed that gluten supplementation significantly inhibited the growth of harmful bacteria, such as *Escherichia coli* and total coliforms, while promoting the growth of *Lactobacillus*, a beneficial genus for gut health. Another surprising finding was that the high-fat diet itself suppressed the growth of these pathogenic bacteria. This novel observation underscores the complex ways in which diets influence the microbiota and calls for further research to understand the mechanisms involved.

The link between gluten intake and weight gain may be driven by a combination of factors, such as impaired thermogenesis, altered gut permeability, and inflammatory pathways. These underlying mechanisms need further exploration in future studies. To counteract the potential weight-gain effects of gluten, prebiotic supplementation might be a viable dietary approach. Therefore, as our study was conducted in animal models, caution is necessary when extending these findings to humans. Future research, both experimental and clinical, should focus on these mechanisms and evaluate how prebiotic supplementation could mitigate gluten’s effects.

## Supplementary Information

Supplementary Information.

## Data Availability

The data that support the findings of this study are not openly available due to reasons of sensitivity and are available from the corresponding author upon reasonable request, provided that the manuscript is accepted and published.

## References

[1] Berg, G. et al. Microbiome definition re-visited: old concepts and new challenges. *Microbiome.***8**, 103. 10.1186/s40168-020-00875-0 (2020).32605663 10.1186/s40168-020-00875-0PMC7329523

[2] Hillman, E. T., Lu, H., Yao, T. & Nakatsu, C. H. Microbial ecology along the Gastrointestinal tract. *Microbes Environ.***32**, 300–313. 10.1264/jsme2.ME17017 (2017).29129876 10.1264/jsme2.ME17017PMC5745014

[3] Palmas, V. et al. Gut microbiota markers associated with obesity and overweight in Italian adults. *Sci. Rep.***11** (1), 1–14. 10.1038/s41598-021-84928-w (2021).33750881 10.1038/s41598-021-84928-wPMC7943584

[4] Hiel, S. et al. Link between gut microbiota and health outcomes in inulin-treated obese patients: lessons from the Food4Gut multicenter randomized placebo-controlled trial. *Clin. Nutr.***39** (12), 3618–3628. 10.1016/j.clnu.2020.04.005 (2020).32340903 10.1016/j.clnu.2020.04.005

[5] Zhang, L. et al. Gut microbiota and therapy for obesity and type 2 diabetes. *Front. Endocrinol. (Lausanne)*. **26**, 15:1333778. 10.3389/fendo.2024.1333778 (2024).10.3389/fendo.2024.1333778PMC1100208338596222

[6] Miranda, V. P. N. et al. do Abundance of Gut Microbiota, Concentration of Short-Chain Fatty Acids, and Inflammatory Markers Associated with Elevated Body Fat, Overweight, and Obesity in Female Adolescents. Mediators Inflamm. 2019:7346863 (2019). 10.1155/2019/734686310.1155/2019/7346863PMC694287931933541

[7] Afzaal, M. et al. Human gut microbiota in health and disease: unveiling the relationship. *Front. Microbiol.***26**, 13:999001. 10.3389/fmicb.2022.999001 (2022).10.3389/fmicb.2022.999001PMC954925036225386

[8] McAllan, L. et al. Protein quality and the protein to carbohydrate ratio within a high fat diet influences energy balance and the gut microbiota in C57BL/6J mice. *PLoS One*. **9** (2), e88904. 10.1371/journal.pone.0088904 (2014).24520424 10.1371/journal.pone.0088904PMC3919831

[9] Ijaz, M. U. et al. Beef, casein, and soy proteins differentially affect lipid metabolism, triglycerides accumulation and gut microbiota of high-fat diet-fed C57BL/6J mice. *Front. Microbiol.***9**, 2200. 10.3389/fmicb.2018.02200 (2018).30319558 10.3389/fmicb.2018.02200PMC6165900

[10] Menta, P. L. R. et al. Wheat gluten intake increases the severity of experimental colitis and bacterial translocation by weakening of the proteins of the junctional complex. *Br. J. Nutr.***121** (4), 361–373. 10.1017/S0007114518003422 (2019).30554574 10.1017/S0007114518003422

[11] Olivares, M. et al. The Janus face of cereals: Wheat-Derived prebiotics counteract the detrimental effect of gluten on metabolic homeostasis in mice fed a high fat/high sucrose diet. *Mol. Nutr. Food Res.***63** (24), 1900632. 10.1002/mnfr.201900632 (2019).31608562 10.1002/mnfr.201900632PMC7003472

[12] Hansen, L. B. S. et al. A low-gluten diet induces changes in the intestinal Microbiome of healthy Danish adults. *Nat. Commun.***9** (1), 4630. 10.1038/s41467-018-07019-x (2018).30425247 10.1038/s41467-018-07019-xPMC6234216

[13] Rinninella, E. et al. el al. Food Components and Dietary Habits: Keys for a Healthy Gut Microbiota Composition, Nutrients. 11(10), 2393 (2019). 10.3390/nu1110239310.3390/nu11102393PMC683596931591348

[14] Freire, R. H. et al. Wheat gluten intake increases weight gain and adiposity associated with reduced thermogenesis and energy expenditure in an animal model of obesity. *Int. J. Obes.***40** (3), 479–486. 10.1038/ijo.2015.204 (2015).10.1038/ijo.2015.20426443339

[15] Aguilar, E. C. et al. Dietary gluten worsens hepatic steatosis by increasing inflammation and oxidative stress in ApoE–/– mice fed a high-fat diet. *Food Funct.***3** (7), 3332–3347. 10.1039/d3fo00149k (2023).10.1039/d3fo00149k36940107

[16] Garcia-Mazcorro, J. F., Noratto, G. & Remes-Troche, J. M. The effect of Gluten-Free diet on health and the gut microbiota cannot be extrapolated from one population to others. *Nutrients***4** (10), 1421. 10.3390/nu10101421 (2018).10.3390/nu10101421PMC621291330287726

[17] Atılgan, S. Determination of Probiotic Potentials of Kefir Produced with Kefir Grain and Kefir Starter Culture as In Vivo Using Qpcr. Master’s Thesis, S.D.U. Institute of Science, Isparta, (2018).

[18] Ren, Z. et al. Gut microbiota-CRAMP axis shapes intestinal barrier function and immune responses in dietary gluten‐induced enteropathy. *EMBO Mol. Med.***14** (8), e14059. 10.15252/emmm.202114059 (2021).10.15252/emmm.202114059PMC835090134125490

[19] Galipeau, H. J. et al. Intestinal microbiota modulates gluten-induced immunopathology in humanized mice. *Am. J. Pathol.***185** (11), 2969–2982. 10.1016/j.ajpath.2015.07.018 (2015).26456581 10.1016/j.ajpath.2015.07.018PMC4630176

[20] Freitag, T. L. et al. Gliadin nanoparticles induce immune tolerance to Gliadin in mouse models of Celiac disease. *Gastroenterol***158** (6), 1667–1681e12. 10.1053/j.gastro.2020.01.045 (2020).10.1053/j.gastro.2020.01.045PMC719835932032584

[21] Mahmood, I. Application of allometric principles for the prediction of pharmacokinetics in human and veterinary drug development. Adv Drug Deliv Rev. 30;59(11):1177-92 (2007). 10.1016/j.addr.2007.05.01510.1016/j.addr.2007.05.01517826864

[22] Swiątecka, D., Złotkowska, D., Markiewicz, L. H., Szyc, A. M. & Wróblewska, B. Impact of Whey proteins on the systemic and local intestinal level of mice with diet induced obesity. *Food Funct.***8** (4), 1708–1717. 10.1039/c6fo01311b (2017).28382342 10.1039/c6fo01311b

[23] Arı, Z. et al. The effect of dehydroepiandrosterone sulfate on oxidant status markers and copper and zinc levels in Hind leg muscle of rats fed with a Fat-Containing diet. *Turk. J. Biochem.* **33** (1), 1–8 (2008).

[24] Halkman, A. K. *Food Microbiology Applications* (Başak Printing and Promotion Services, 2005).

[25] Hitchins, A. D., Feng, P., Watkins, W. D., Rippey, S. R. & Chandler, L. A. Escherichia coli and the coliform bacteria. In: Tomlinson, L. A. (Ed.), Food and Drug Administrations. 8th edn. Washington, DC: AOAC International. p. (2000). 4.01–4.29.

[26] US Food and Drug Administration (FDA). Bacteriological Analytical Manual (BAM). (2001). Available from: http://www.cfsan.fda.gov/~ebam/bam-toc.html

[27] Bektaş, A., Özel, M. & Gluten Friend or foe?? *Curr. Gastroenterol.* **22** (2), 127–134 (2018).

[28] Sanz, Y. Effects of a gluten-free diet on gut microbiota and immune function in healthy adult humans. *Gut Microbes*. **1** (3), 135–137. 10.4161/gmic.1.3.11868 (2010).21327021 10.4161/gmic.1.3.11868PMC3023594

[29] De Palma, G., Nadal, I., Collado, M. C. & Sanz, Y. Effects of a gluten-free diet on gut microbiota and immune function in healthy adult human subjects. *Br. J. Nutr.***102** (8), 1154–1160. 10.1017/S0007114509371767 (2009).19445821 10.1017/S0007114509371767

[30] Rune, I. et al. Modulating the gut microbiota improves glucose tolerance, lipoprotein profile and atherosclerotic plaque development in ApoE-Deficient mice. *PLoS ONE*. **11** (1), e0146439. 10.1371/journal.pone.0146439 (2016).26799618 10.1371/journal.pone.0146439PMC4723129

[31] Seguella, L. et al. High-fat diet impairs duodenal barrier function and elicits glia-dependent changes along the gut-brain axis that are required for anxiogenic and depressive-like behaviors. **16**;18(1):115 (2021). 10.1186/s12974-021-02164-510.1186/s12974-021-02164-5PMC812615833993886

[32] Te Morenga, L., Mallard, S. & Mann, J. Dietary sugars and body weight: systematic review and meta-analyses of randomised controlled trials and cohort studies. *BMJ***346**, e7492. 10.1136/bmj.e7492 (2012).23321486 10.1136/bmj.e7492

[33] Troncone, R. et al. Effects of gluten enriched diet on the small intestinal mucosa of normal mice and mice with graft versus host reaction. *Gut***35** (6), 779–782. 10.1136/gut.35.6.779 (1994).8020805 10.1136/gut.35.6.779PMC1374878

[34] Liang, T. T. et al. Wheat gluten regulates cholesterol metabolism by modulating gut microbiota in hamsters with hyperlipidemia. *J. Oleo Sci.***68** (9), 909–922. 10.5650/jos.ess18257 (2019).31484903 10.5650/jos.ess18257

[35] Zhao, F., Song, S., Xu, X., Zhou, G. & Li, C. A short-term feeding of dietary casein increases abundance of Lactococcus Lactis and upregulates gene expression involving obesity prevention in cecum of young rats compared with dietary chicken protein. *Front. Microbiol.***10**, 2411. 10.3389/fmicb.2019.02411 (2019).31708891 10.3389/fmicb.2019.02411PMC6824296

[36] Wei, C. et al. High-fat diet disrupts the gut microbiome, leading to inflammation, damage to tight junctions, and apoptosis and necrosis in Nyctereutes procyonoides intestines. *Microbiol. Spectr.***20** (4), e04182–e04123. 10.1128/spectrum.04182-23 (2024).10.1128/spectrum.04182-23PMC1098659738376358

[37] Casadesus, J. & Urdaneta, V. Interactions between Bacteria and bile salts in the Gastrointestinal and hepatobiliary tracts. *Front. Med. (Lausanne)*. **3**, 4:163. 10.3389/fmed.2017.00163 (2017).10.3389/fmed.2017.00163PMC563235229043249

