# Supplementary material for: Effects of dietary gluten on body weight and gut microbiota in BALB-C mice using 16 S rRNA-Based analysis
Source: Sci Rep. 2025 Mar 7;15:7959. doi: 10.1038/s41598-025-92213-3 (PMC11889222; doi:10.1038/s41598-025-92213-3)
Supplement: Supplementary file 1 — Supplementary Information. [file 41598_2025_92213_MOESM1_ESM.docx]

**Supplementary Information for Effects of dietary gluten on body weight and gut microbiota in BALB-C mice using 16S rRNA-Based analysis**

**Supplementary Table S1.** Body Weight Change by Diet Groups

|  | Diet Groups | N | Mean | ss | F | p-value | Difference |
| --- | --- | --- | --- | --- | --- | --- | --- |
| Body  Weight  Change | HFD | 6 | 10,272 | 0,430 | 146,581 | **p<0.05*** | 3<1 (**p<0.05**)** 3<4 (**p<0.05**)**  3<2 (**p<0.05**)**  4<2 (**p<0.05**)**  1<2 (**p<0.05**)** |
|  | HFD-G | 6 | 12,265 | 0,680 |  |  |  |
|  | SD | 6 | 6,858 | 0,205 |  |  |  |
|  | SD-G | 6 | 9,151 | 0,531 |  |  |  |
| **: ANOVA; **: LSD, (levene Statistic:2,288, p=0,104) 1: HFD (high-fat diet),2: HFD-G (: high-fat diet with gluten), 3:SD (standard diet), 4:SD-G (standard diet with gluten). N: Sample size, F: Fisher’s Statistic, ss: Sum of squares, p:p-value, significance level set at p<0.05. Statistically significant p-values (<0.05) are indicated in bold.*  **Supplementary Table S2.** Total Mesophilic Aerobic Bacteria Change by Diet Groups   \|  \| Diet Groups \| N \| Mean \| ss \| F^a^ \| p-value \| Difference \| \| --- \| --- \| --- \| --- \| --- \| --- \| --- \| --- \| \| Total Mesophilic Aerobic Bacteria \| HFD \| 6 \| -60662,05 \| 177771,83 \| 1,969 \| 0,546* \| - \| \| HFD-G \| 6 \| -63774,41 \| 338141,03 \| \| SD \| 6 \| -126626,11 \| 281751,06 \| \| SD-G \| 6 \| -226307,00 \| 77037,55 \| \| **: WELCH, (levene Statistic:7,413, p=0,001), ^a^:Asymptotically F, 1: HFD (high-fat diet),2: HFD-G (: high-fat diet with gluten), 3:SD (standard diet), 4:SD-G (standard diet with gluten). N: Sample size, F: Fisher’s Statistic, ss: Sum of squares, p:p-value, significance level set at p<0.05. Statistically significant p-values (<0.05) are indicated in bold.* \| \| \| \| \| \| \| \|   **Supplementary Table S3.** *Lactobasillus spp.* Change by Diet Groups   \|  \| Diet  Groups \| N \| Rank \| χ² \| p-value \| Difference \| \| --- \| --- \| --- \| --- \| --- \| --- \| --- \| \| *Lactobasillus* \| HFD \| 6 \| 6,86 \| 21,806 \| **p<0.05*** \| 1<2 (z=-3,130, **p<0.05**)** 3<4 (z=-3,030, **p<0.05**)** \| \| HFD-G \| 6 \| 24,14 \| \| SD \| 6 \| 8,14 \| \| SD-G \| 6 \| 18,86 \| \|  \| **: Kruskal Wallis Test **: Mann-Whitney U 1: HFD (high-fat diet),2: HFD-G (: high-fat diet with gluten), 3:SD (standard diet), 4:SD-G (standard diet with gluten). N: Sample size, χ²: chi-squared, z: z-score, p:p-value, significance level set at p<0.05. Statistically significant p-values (<0.05) are indicated in bold.* \| \| \| \| \| \|   **Supplementary Table S4.** Total Coliform Group Bacteria Change by Diet Groups   \|  \| Diet  Groups \| N \| Mean \| ss \| F \| p \| Difference \| \| --- \| --- \| --- \| --- \| --- \| --- \| --- \| --- \| \| Total Coliform Group Bacteria \| HFD \| 6 \| -1114482,65 \| 196080,80 \| 19,823 \| **p<0.05*** \| 1<3 (**p<0.05**)** 4<3 (**p<0.05**)**  2<1 (p>0.05)** \| \| HFD-G \| 6 \| -1189457,96 \| 251367,95 \| \| SD \| 6 \| -389963,02 \| 227084,85 \| \| SD-G \| 6 \| -618584,22 \| 240728,35 \| \| **: ANOVA **: LSD, (levene Statistic:0,466,p=0,709), 1: HFD (high-fat diet),2: HFD-G (: high-fat diet with gluten), 3:SD (standard diet), 4:SD-G (standard diet with gluten). N: Sample size, F: Fisher’s Statistic, ss: Sum of squares, p:p-value, significance level set at p<0.05. Statistically significant p-values (<0.05) are indicated in bold.* \| \| \| \| \| \| \| \|   **Supplementary Table S5.** *Escherichia coli* Change by Diet Groups   \|  \| Diet  Groups \| \| N \| Rank \| χ² \| p \| Difference \| \| --- \| --- \| --- \| --- \| --- \| --- \| --- \| --- \| \| *Escherichia coli* \| HFD \| \| 6 \| 11,71 \| 17,583 \| **p<0.05*** \| 1<3 (z=-2,875, **p<0.05**)** 4<3 (z=-0,831, **p=0,456**)**  2<1 (z=-2,236, **p<0.05**)** \| \| HFD-G \| \| 6 \| 5,29 \| \| SD \| \| 6 \| 22,14 \| \| SD-G \| \| 6 \| 18,86 \| \|  \| \| **: Kruskal Wallis Test **: Mann-Whitney U 1: HFD (high-fat diet),2: HFD-G (: high-fat diet with gluten), 3:SD (standard diet), 4:SD-G (standard diet with gluten). N: Sample size, χ²: chi-squared, z: z-score, p:p-value, significance level set at p<0.05. Statistically significant p-values (<0.05) are indicated in bold.* \| \| \| \| \| \| | | | | | | | |
